# RNA-Seq Analysis Unraveling Novel Genes and Pathways Influencing Corneal Wound Healing

**DOI:** 10.1167/iovs.65.11.13

**Published:** 2024-09-06

**Authors:** Rajnish Kumar, Ratnakar Tripathi, Nishant R. Sinha, Rajiv R. Mohan

**Affiliations:** 1 Harry S. Truman Memorial Veterans’ Hospital, Columbia, Missouri, United States; 2 Department of Veterinary Medicine & Surgery, College of Veterinary Medicine, University of Missouri, Columbia, Missouri, United States; 3 Department of Ophthalmology, School of Medicine, University of Missouri, Columbia, Missouri, United States

**Keywords:** cornea, fibrosis, RNA-seq, sequencing, wound healing

## Abstract

**Purpose:**

Transdifferentiation of corneal fibroblasts to myofibroblasts in the stroma is a central mechanistic event in corneal wound healing. This study sought to characterize genes and pathways influencing transdifferentiation of human corneal fibroblasts (hCSFs) to human corneal myofibroblasts (hCMFs) using RNA sequencing (RNA-seq) to develop comprehensive mechanistic information and identify newer targets for corneal fibrosis management.

**Methods:**

Primary hCSFs were derived from donor human corneas. hCMFs were generated by treating primary hCSFs with transforming growth factor β1 (TGFβ1; 5 ng/mL) for 72 hours under serum-free conditions. RNA was extracted using the RNeasy Plus Mini Kit and subjected to RNA-seq analysis after quality control testing. Differential gene expression, pathway enrichment, and protein–protein network analyses were performed using DESeq2, GSEA/PANTHER/Reactome, and Cytoscape/cytoHubba, respectively.

**Results:**

RNA-seq analysis of hCMFs and hCSFs identified 3843 differentially expressed genes and transcripts (adjusted *P* < 0.05). The log(fold change) ≥ ±1.5 filter showed 816 upregulated and 739 downregulated genes between two cell types. Pathway enrichment analysis showed the highest normalized enrichment score for epithelial-to-mesenchymal transition (5.569), followed by mTORC1 signaling (2.949), angiogenesis (2.176), and TGFβ signaling (2.008). Protein–protein interaction network analysis identified the top 20 nodes influencing corneal myofibroblast development. The expression of a novel MXRA5 in corneal stroma and its association with corneal fibrosis was verified by real-time quantitative reverse transcription PCR and immunofluorescence. RNA-seq and gene count files were submitted to the NCBI Gene Expression Omnibus (GSE260476).

**Conclusions:**

This study identified several novel genes involved in myofibroblast development, offering potential targets for developing newer therapeutic strategies for corneal fibrosis.

The cornea, an outermost transparent, avascular, and immune-privileged tissue of the eye, contributes two-thirds of refraction and protection to eyes.^1^ An uncontrolled corneal healing after trauma or injury often leads to corneal scarring or fibrosis and vision loss in millions of people worldwide.^2^ About 1.5 million new cases of corneal haze or fibrosis are recorded each year, and nearly 4% Americans currently endure corneal blindness.^3^^–^^5^

The stroma constitutes 90% of the cornea and contains keratocytes, collagens, extracellular matrix (ECM), and proteoglycans.^6^ Keratocytes, the quiescent transparent cells, play an important role in corneal homeostasis and wound healing.^7^ Keratocytes transdifferentiate into myofibroblasts under the influence of cytokines and facilitate tissue repair primarily after trauma or injury. Transforming growth factor β1 (TGFβ1), among many cytokines, has been shown to play a major role in the generation of myofibroblasts, which are characterized by de novo alpha-smooth muscle actin (αSMA) and collagen (Col) I and Col III proteins. Corneal stromal myofibroblasts express high amounts of intermediate filaments, fibronectin, vimentin, and desmin at the early stages of transdifferentiation.^8^ Inadequate reductions and the persistence of corneal myofibroblasts in stroma after wound closure leads to corneal scarring and fibrosis and abnormal stromal ECMs,^9^^–^^11^ and reduced crystallin alters the refractive index and light-scattering property of the cornea.^12^

High-throughput next-generation RNA sequencing (RNA-seq) and bioinformatics analyses allow identification of differentially expressed genes (DEGs), hub genes, and signaling pathways associated with corneal scar development.^13^ This technique is particularly useful for unraveling molecular complexities and discovering prospective targets for corneal scar treatment. This study examined transcriptional differences between human corneal stromal fibroblasts (hCSFs) and TGFβ1-induced fibrotic human corneal myofibroblasts (hCMFs) using RNA-seq and bioinformatics analyses.

## Methods

### Primary Culture of Human Corneal Stromal Cells

Healthy donor human corneas (three males and one female, 49–76 years of age) obtained from Saving Sight (Kansas City, MO, USA) and normal (non-fibrotic) and fibrotic cadaver corneas from human subjects were used. The use of such corneas does not constitute human subjects research according to the Department of Health and Human Services regulatory definitions. Primary hCSFs were generated by rinsing corneas with serum-free Minimum Essential Medium (MEM), removing the corneal endothelium and epithelium with a surgical scalpel blade, cutting them into small fragments, and incubating at 37°C in a 5% CO_2_ incubator for 3 to 5 weeks in MEM supplemented with 10% heat-inactivated fetal bovine serum (Thermo Fisher Scientific, Waltham, MA, USA).

### Differentiation of Fibroblasts to Myofibroblasts

The corneal fibrosis, in vitro, was initiated by inducing transdifferentiation of hCSFs into hCMFs by growing cultures in serum-free conditions for 8 hours followed by TGFβ1 (5 ng/mL) (PeproTech, Cranbury, NJ, USA) exposure under serum-free MEM for 72 hours. Cultures were fed with fresh media and TGFβ1 every 24 hours.

### Immunofluorescence

Double immunofluorescence was used to verify levels of fibroblast-specific protein 1 (FSP1; a fibroblast marker) and αSMA (a myofibroblast marker) in hCSFs and hCMFs using a mouse monoclonal anti-αSMA (M0851, diluted 1:100; Dako, Carpinteria, CA, USA) and FSP1 (ab41532, diluted 1:100; Abcam, Cambridge, UK) antibodies. The staining protocol involved fixation of cells with 4% freshly prepared paraformaldehyde followed by blocking with 5% BSA in phosphate-buffered saline (PBS) plus 0.01% Tween 20 for 1 hour at room temperature and incubation in primary anti-αSMA and anti-FSP1 antibodies at 4°C overnight. Cells were washed with PBS containing 0.05% Tween 20 (PBST; three times for 10 minutes each) followed by incubation in Invitrogen Goat anti-Mouse IgG (H+L) Highly Cross-Adsorbed Secondary Antibody, Alexa Fluor 488 (A-11029, diluted 1:500; Thermo Fisher Scientific) and Invitrogen Goat anti-Rabbit IgG (H+L) Cross-Adsorbed Secondary Antibody, Alexa Fluor 594 (A-11012, diluted 1:500; Thermo Fisher Scientific) antibodies for 1 hour at room temperature. They were then washed with PBST three times (10 minutes each) and mounted in VECTASHIELD containing 4′,6-diamidino-2-phenylindole (DAPI; Vector Laboratories, Newark, CA, USA). A Leica DM4000 B fluorescence microscope (Leica Microsystems, Wetzlar, Germany) was used for visualization.

### RNA Extraction and RNA-Seq

Primary hCSF and hCMF cultures were used in quadruplicate. To obtain RNA, cultures were first washed with 1× PBS, scraped with a “rubber policeman” in lysis buffer and subjected to the RNeasy Mini Kit (QIAGEN, Hilden, Germany) following the manufacturer's instructions. Isolated total RNA was reverse transcribed to cDNA using a commercial kit (Promega Corporation, Madison WI, USA) following the manufacturer's instructions. The reaction mixture was heated at 42°C for 30 minutes followed by enzyme inactivation at 90°C for 2 minutes.^14^ The RNA samples were sequenced by Arraystar (Rockville, MD, USA), and cDNAs were used for real-time quantitative reverse transcription PCR (qRT-PCR).

### Differential Expression Analysis

**Figure 1.**
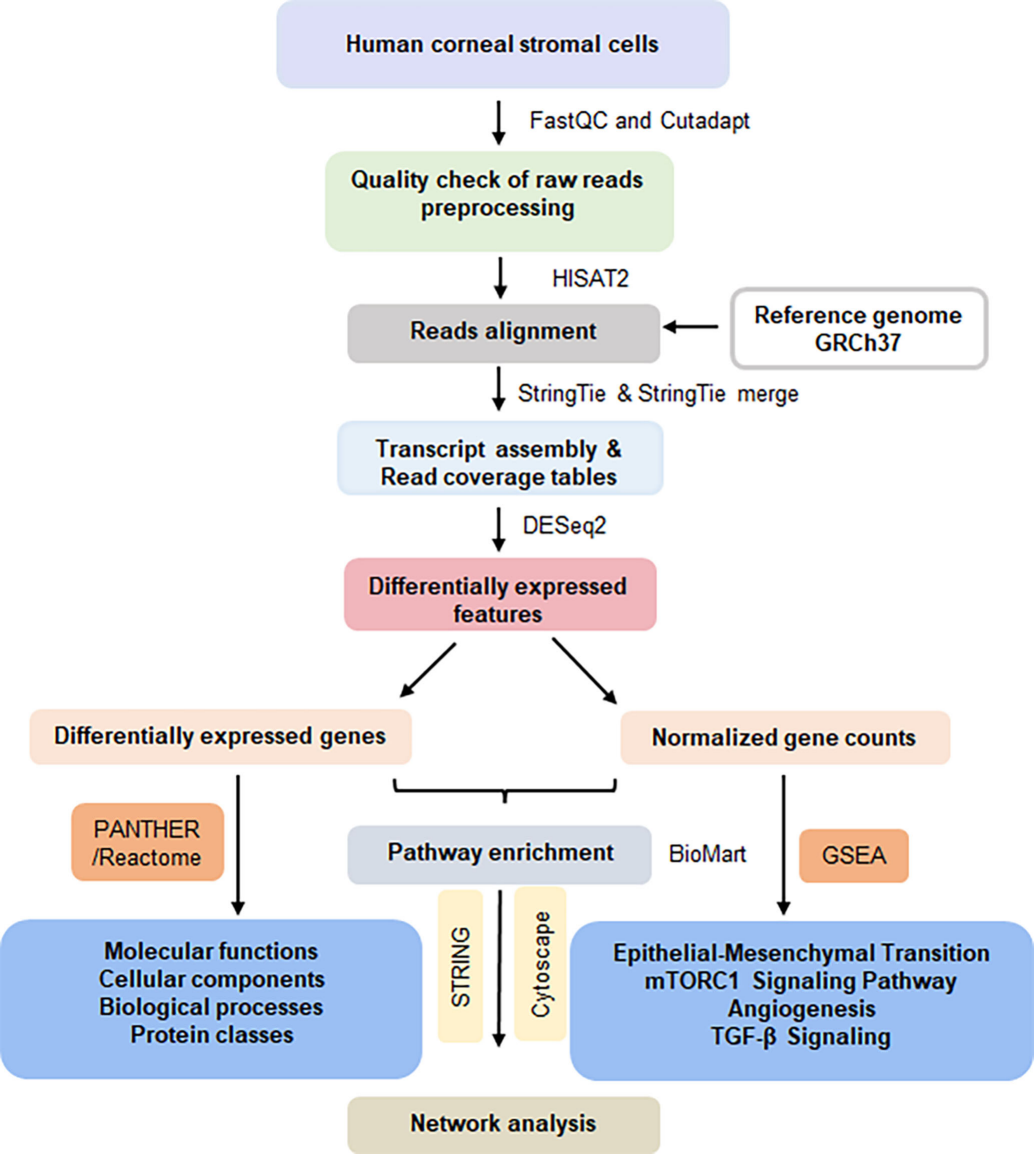
Schematic representation of the methodology adopted in this study for RNA-seq data analysis.

The quadruplet samples of hCSF and hCMF were subjected to differential gene expression (DGE) analysis using a published method^15^ and employing Python and R packages (R Foundation for Statistical Computing, Vienna, Austria) and bioinformatics tools. Figure 1 shows the approach utilized in this study. FastQC 0.12.1^16^ and Cutadapt 4.6^17^ were used for quality checks and to filter out adapter sequences, primers, poly-A tails, and other types of unwanted sequences from the high-throughput sequencing reads. We evaluated the quality of all eight RNA sequences from the four hCSF samples and four hCMF samples, and unwanted sequences were removed to ensure high-quality reads during the quality check. HISAT2^18^ was used for read-alignment of trimmed RNA sequences of both the hCSF and hCMF cell types with the human reference genome GRCh37. HISAT2 utilizes a novel genome indexing scheme by implementing a graph-based approach to capture a wide representation of genetic variant reads. HISAT2 implements the Graph FM (GFM) index, unlike other graph aligner methods (e.g., vg19, bpa aligner20).^19^ StringTie 2.2.1^20^ was used for assembling mapped reads into transcripts via a network flow algorithm and optional de novo assembly. StringTie creates comprehensive and accurate gene reconstructions and precise estimations of expression levels compared to other prominent transcript assembly tools, such as Cufflinks,^21^ IsoLasso,^22^ Scripture,^23^ and Traph.^24^ Furthermore, for each sample, StringTie merge was run with all assembled transcript files (in GTF/GFF format), reference annotation files (gencode.v32.annotation.gtf), and a global and unified set of transcripts (isoforms) from various RNA-seq samples. The gene count files obtained from StringTie were used as input for DESeq2 1.34.0 to identify differentially expressed features.^25^ In addition, normalized gene counts were generated for gene set enrichment analysis (GSEA; version 4.3.2).^26^ To add annotation information from the GTF file to the DEGs, Annotate DESeq2/DEXSeq was used.

### Pathway Enrichment Analysis

To distinguish biological processes, cellular components, molecular functions, protein classes, and pathways of DEGs, protein annotation evolutionary relationship (PANTHER 18.0) classification (https://pantherdb.org/) and the Reactome database (https://reactome.org/) were used.^27^ A significance level of *P* < 0.05 was established as the threshold for determining substantial enrichment. To identify key pathways that are enriched in hCMFs compared to hCSFs, GSEA is a useful computational method that determines predefined statistically significant and concordant differences between two biological states (hCMFs and hCSFs). The enriched pathways were organized based on their normalized enrichment scores, and only those with a significance level of *P* < 0.01 were selected for subsequent analysis.

### Protein‒Protein Interaction Networks

To analyze known proteins and protein–protein interaction (PPI) networks, the Search Tool for the Retrieval of Interacting Genes/Proteins (STRING) database and web-based search engine were used. The direct and indirect connections between proteins and their functional correlations were analyzed (https://string-db.org/) using a reported method.^28^ The STRING database was used for creating the corresponding PPI networks from the most enriched pathways obtained from the GSEA. Hub genes were identified based on network topology using the PPI network. Afterward, using Cytoscape 3.8.2 and the cytoHubba plugin, we identified the probable targets and influential nodes inside intricate networks.^29^ The analysis focused on examining the crucial hub genes that may be involved in the process of corneal wound healing.

### Validation of Selected Genes Using qRT-PCR

For qRT-PCR, we used the StepOnePlus Real-Time PCR System (Applied Biosystems, Waltham, MA, USA) and gene-specific primers (Table 1) to validate expression of selected genes. A 20-µL reaction mixture contained 1 µL cDNA, 1 µL forward primer, 1 µL reverse primer, and 10 µL iQ SYBR1 Green Supermix (Bio-Rad Laboratories, Hercules, CA, USA) per reaction well. The mixture was exposed to the following PCR parameters: 95°C for 5 minutes, followed by 40 cycles of 95°C for 15 seconds, then 60°C for 1 minute, and then a final cycle of 72°C for 10 minutes. The fluorescence threshold value (*C_t_*) was calculated to detect signal differences in association with an exponential increase of PCR products in the log-linear phase. The *GAPDH* gene was used for the normalization of data. Relative expression/fold change (FC) over the corresponding values for the control was calculated by the 2^–^^ΔΔCt^ method. Two or three independent experiments were executed; for each sample, qRT-PCR was performed in triplicate, and the average fold changes in mRNA levels were calculated.

**Table 1.** qRT-PCR Primer Sequences

Gene	Protein	Accession No		Primers (5′–3′)
*MXRA5*	Matrix-remodeling associated 5	NM_015419	F	CCACCTTAGCTGGATTCTTC
			R	GACCTTTGGGATGGAAAGAG
*POSTN*	Periostin	NM_001135934	F	ACTGGAGGTGGAGAAACA
			R	GAACGACCTTCCCTTAATCG
COL5A1	Collagen type V alpha 1 chain	NM_000093	F	CGAGGGTGAGACCTATTACT
			R	GGACCTCTGTGGTTTCTTTG
GAPDH	Glyceraldehyde-3-phosphate dehydrogenase	NM_002046	F	TGGGTGTGAACCATGAGA
			R	GTCCTTCCACGATACCAAAG

F, forward; R, reverse.

### Validation of MXRA5 Protein

Immunocytochemistry and immunohistochemistry were used to confirm the expression of MXRA5 protein in non-fibrotic and fibrotic hCSFs and human corneas. A double immunofluorescence was performed as described earlier using the Dako mouse monoclonal anti-αSMA antibody and an Abcam goat polyclonal anti-MXRA5 antibody (ab211302) at 1:100 dilution. Invitrogen Donkey anti-Mouse IgG (H+L) Highly Cross-Adsorbed Secondary Antibody, Alexa Fluor 594 (A-21203; Thermo Fisher Scientific) and Invitrogen Donkey anti-Goat IgG (H+L) Cross-Adsorbed Secondary Antibody, Alexa Fluor 488 (A-11055; Thermo Fisher Scientific) secondary antibodies at 1:500 dilutions were used. Corneal sections were incubated in PBS for 10 minutes followed by blocking with 5% horse serum in PBST (Jackson ImmunoResearch, West Grove, PA, USA) for 1 hour at room temperature, incubation in MXRA5 and αSMA primary antibodies overnight at 4°C, washing with PBST, and incubation with the Invitrogen Donkey anti-Mouse IgG (H+L) Highly Cross-Adsorbed Secondary Antibody, Alexa Fluor 594 (A-21203; Thermo Fisher Scientific) and Invitrogen Donkey anti-Goat IgG (H+L) Cross-Adsorbed Secondary Antibody, Alexa Fluor 488 (A-11055; Thermo Fisher Scientific) secondary antibodies for 1 hour at room temperature, and mounting with the VECTASHIELD mounting medium containing DAPI. The Leica DM4000 B fluorescence microscope equipped with a digital camera system (Spot) was used.

### Statistical Analysis

Statistical analysis was performed using Prism 9.2 (GraphPad, Boston, MA, USA). Student's *t*-test was used to determine statistical significance. The data are presented as the average mean ± standard error (SEM). In statistical analysis, *P* ≤ 0.05 was considered significant.

## Results

### Human Corneal Stromal Fibroblast and Myofibroblast Cells

**Figure 2.**
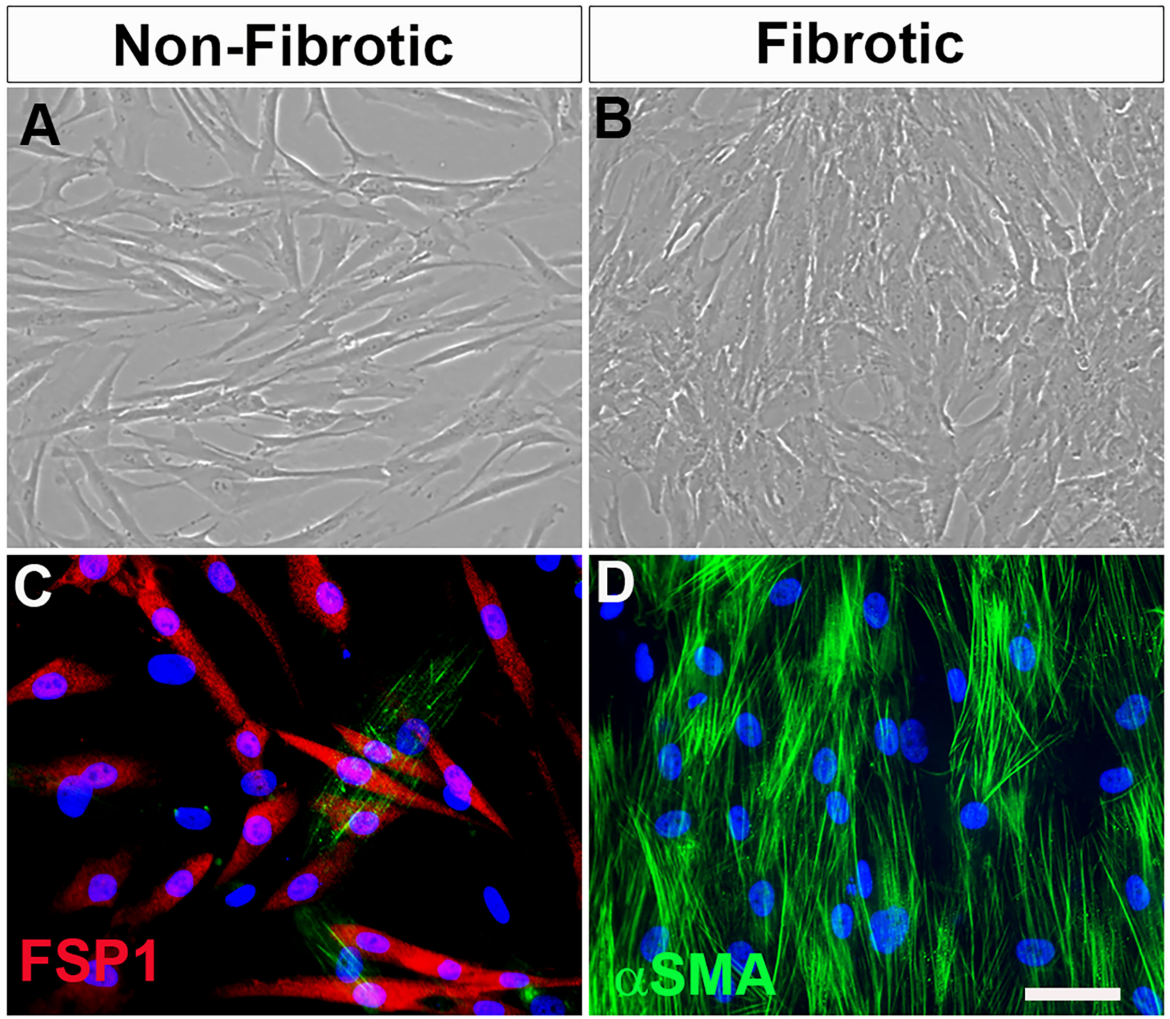
(**A**, **B**) Light microscopy phase-contrast images of non-fibrotic hCSF (**A**) and fibrotic hCMF (**B**) cell types at 72 hours. (**C**, **D**) Double immunofluorescence staining confirms the non-fibrotic and fibrotic characteristics of cell types with FSP1 (**C**) and αSMA (**D**), respectively. *Scale bar*: 20 µm.

The hCSFs showing the fibrotic phenotype transdifferentiated into hCMFs and acquired a myofibroblastic phenotype upon TGFβ stimulation at 72 hours. The phase-contrast microscopy and double immunofluorescence confirmed fibroblastic features (non-fibrotic cells) (Figs. 2A, 2C) and myofibroblastic features (fibrotic cells) (Figs. 2B, 2D).

### Qualitative and Quantitative Analyses of RNA Samples

The high purity and yield of all RNA samples extracted from hCMFs and hCSFs are shown in Table 2. The base call accuracy was 99.9% as per the Phred quality score (*Q* > 30) (Supplementary Fig. S1).

**Table 2.** RNA Sample Preparation Details for hCSF and hCMF Samples Using the RNeasy Mini Kit

Sample ID	Cell Type	Media	Treatment	Harvest Time (hr)	Cellular Phenotype	RNA Yield (ng/µL)	A260/280
hCSF-72h-A1	hCSF	MEM	None	72	Fibroblasts	317.6	2.12
hCSF-72h-A2	hCSF	MEM	None	72	Fibroblasts	414.4	2.11
hCSF-72h-A3	hCSF	MEM	None	72	Fibroblasts	423.6	2.11
hCSF-72h-A4	hCSF	MEM	None	72	Fibroblasts	308.1	2.11
hCMF-72h-B1	hCMF	MEM	TGFβ1 (5 ng/mL)	72	Myofibroblast	256.5	2.09
hCMF-72h-B2	hCMF	MEM	TGFβ1 (5 ng/mL)	72	Myofibroblast	363	2.11
hCMF-72h-B3	hCMF	MEM	TGFβ1 (5 ng/mL)	72	Myofibroblast	391.9	2.11
hCMF-72h-B4	hCMF	MEM	TGFβ1 (5 ng/mL)	72	Myofibroblast	481.7	2.12

MEM, minimum essential media; A260/280, absorption ratio between 260 nm (nucleic acids) and 280 nm (proteins).

### Differential Gene Expression Analysis

**Figure 3.**
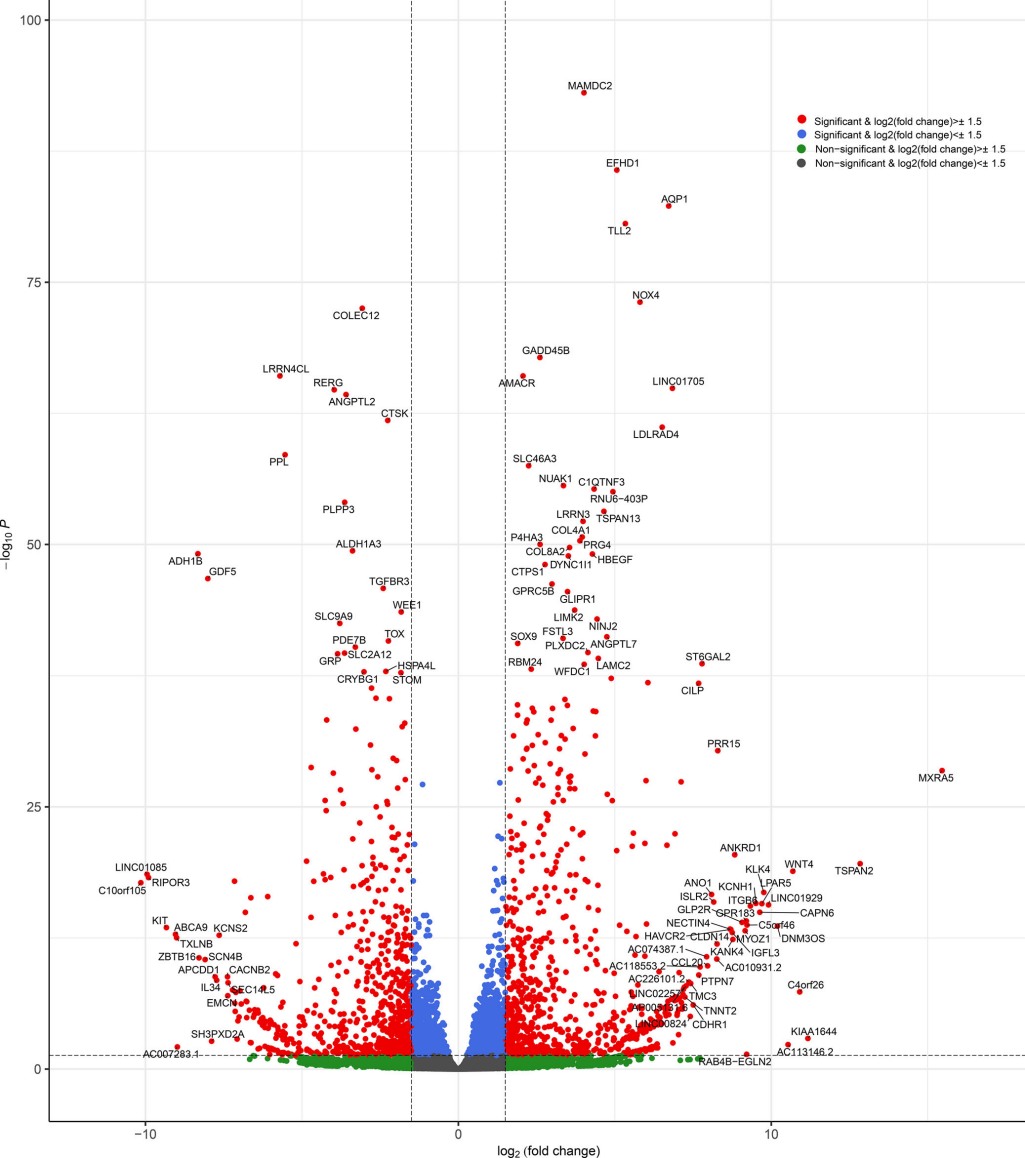
Volcano plot illustrating differential gene expression in the hCMF and hCSF cell types. This plot highlights significantly altered genes, which were upregulated and downregulated (in *red*) at a significance level of *P* ≤ 0.05 and a cutoff value of log_2_(FC) ± 1.5. The non-significant genes are depicted in *gray* and *green*, and genes outside the log_2_(FC) cutoff values are shown in *blue*. The plot distinctly shows the differential expression patterns between the hCMF and hCSF cell types, emphasizing the genes that are notable outliers in their expression levels.

**Figure 4.**
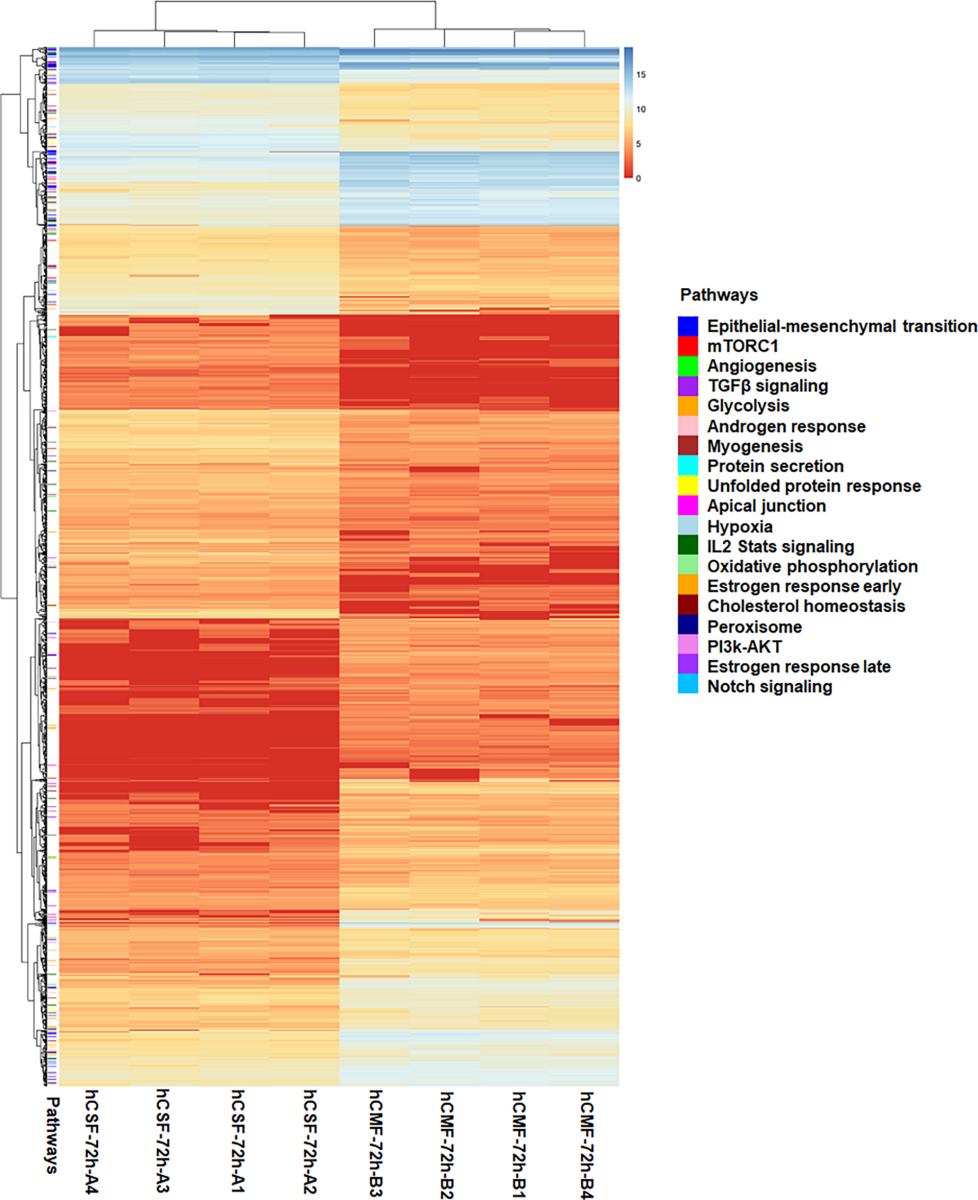
Heatmap of gene expression differences between the hCMF and hCSF sequence profiles. This heatmap illustrates the expression levels of the genes with significant differential expression between the hCMF and hCSF sequences. The genes are displayed as rows and the hCMFs and hCSFs are shown as columns; a *blue*–*yellow*–*red* gradient is used to indicate the expression intensity. *Blue* represents higher expression, and *red* denotes lower expression. The heatmap also indicates key pathways associated with the DEGs.

Table 3 shows the results of RNA-seq read alignment and mapping with the human reference genome (GRCh37) determined by the HISAT2. The 20,269 gene annotations were identified by StringTie and subjected to DESeq2 to obtain DEGs (hCMF vs. hCSF). The changes in gene expression between hCMFs and hCSFs indicated by the *MA* plot for the *M* values, log_2_(FC), and *A* values (average expressions) are shown in Supplementary Fig. S2A, and the violin plot (Supplementary Fig. S2B) unveiled differences in gene expression levels. A total of 3843 significantly expressed genes were found with *P* ˂ 0.05 (Supplementary Table S1). Cutoff values of log_2_(FC) ≥ 1.5 and log_2_(FC) ≤ –1.5 for upregulated and downregulated genes, respectively, led to the identification of 816 upregulated and 739 downregulated genes. The volcano plot shows that genes changed significantly (red, *P* ≤ 0.05), non-significantly (gray), and outside the cutoff values (blue, log_2_[FC]; green, *P*) (Fig. 3). The heatmap of the blue–yellow–red color gradient (blue for highest and red for lowest) demonstrates the expression of DEGs in hCMFs and hCSFs (Fig. 4).

**Table 3.** Mapping Summary of the Eight Sequence Samples

Samples	Raw Pairs	Trimmed	mtRNAs^*^	rRNAs^†^	Mapped
hCSF-72h-A1	16632504	16632498	3.21%	0.71%	89.71%
hCSF-72h-A2	18364824	18364819	1.20%	0.37%	85.83%
hCSF-72h-A3	15389485	15237684	2.82%	0.62%	92.08%
hCSF-72h-A4	15509505	15205190	1.94%	0.51%	90.51%
hCMF-72h-B1	21662055	21662051	3.14%	0.64%	88.60%
hCMF-72h-B2	17617386	17617384	1.41%	0.30%	90.29%
hCMF-72h-B3	21123119	21123117	2.63%	0.52%	91.07%
hCMF-72h-B4	25953221	25953219	1.51%	0.51%	84.06%

The raw sequencing fragment numbers (read pairs) are shown as raw pairs, and fragment numbers after 5′,3′-adapter trimmed and filtered <20-bp reads are shown as trimmed. The percentage of reads number aligned and not aligned to reference genome in trimmed pairs are shown as mapped and unmapped, respectively.

* mtRNAs, the percentage of mitochondrial RNA fragments (read pairs) in trimmed pairs.

† rRNA, the percentage of ribosomal RNA fragments (read pairs) in trimmed pairs.

### Pathway Enrichment Analysis

**Figure 5.**
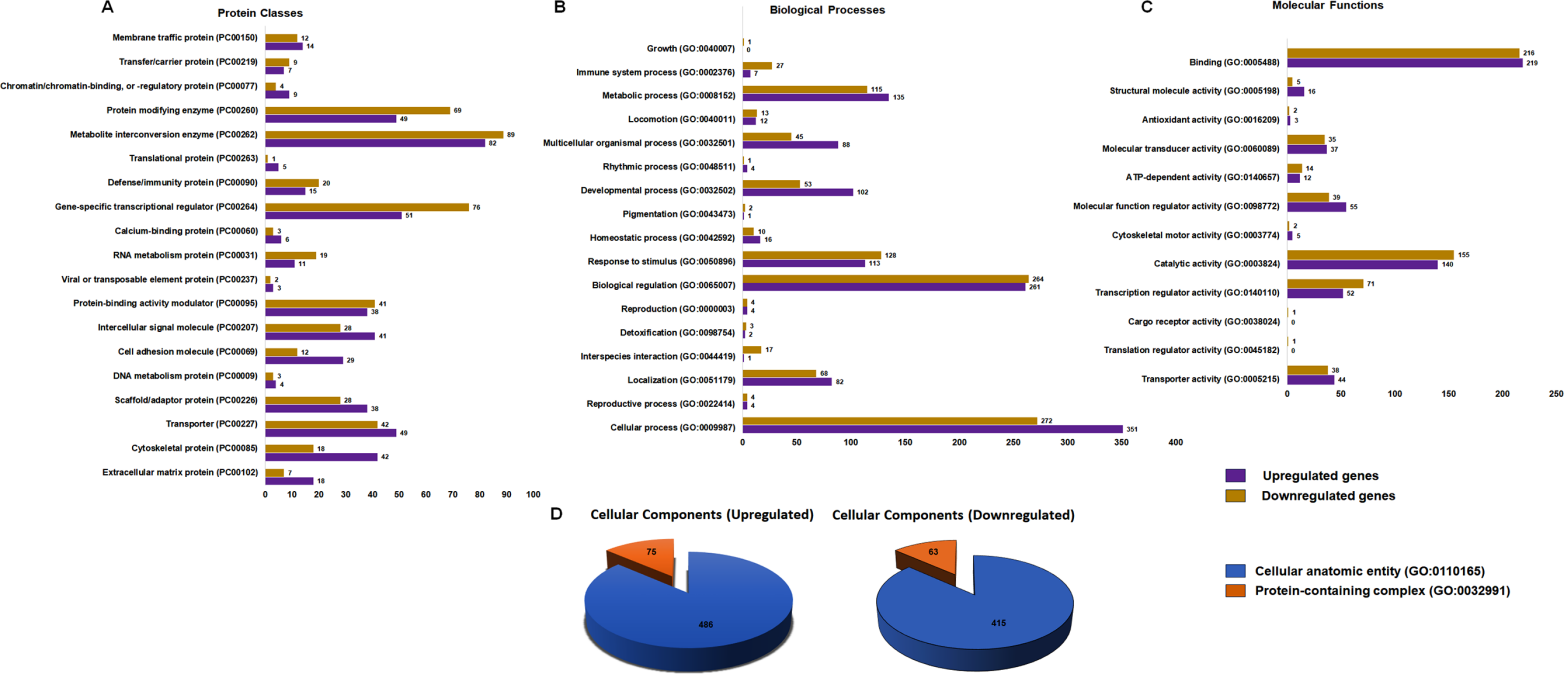
Comprehensive PANTHER analysis of pathway enrichment using upregulated and downregulated genes in hCMFs compared to hCSFs. (**A**) The distribution of protein classes among the DEGs, distinguishing between those that were upregulated and those that were downregulated in hCMFs. (**B**) Biological processes used to categorize the genes highlight the variations in process participation between upregulated and downregulated genes in hCMFs. (**C**) Molecular function analysis of the genes at the molecular level, illustrating the functional differences in the number of genes in hCMFs. (**D**) Cellular component analysis showing the components in which these genes were active, highlighting the distinctions in cellular localization between upregulated and downregulated genes. Each category is presented in a format that allows for easy comparison of the functional and biological attributes associated with gene expression changes in hCMFs compared to hCSFs.

The biological relevance of 816 up- and 739 downregulated genes was scored by the PANTHER pathway enrichment classification system. Figure 5 shows the observed distributions of protein classes (Fig. 5A), variations in biological processes (Fig. 5B), differences in molecular functions (Fig. 5C), and cellular components (Fig. 5D) for the hCMFs and hCSFs. The upregulated genes in hCMFs belonged to protein classes pertaining to the cytoskeletal (42 against 18 genes), cell adhesion (29 against 12 genes), intracellular signaling proteins (41 against 28 genes), ECM (18 against seven genes), adaptor (38 against 28 genes), and translational (five against one gene). On the other hand, downregulated genes in hCMFs were in the protein classes of transcriptional regulator (51 against 76 genes), protein modifying enzyme (49 against 69 genes), and RNA metabolism (11 against 19 genes). The mapping of up- and downregulated genes was performed to understand the contributions of different biological processes in corneal microenvironment (Fig. 5B). Differences in the number of augmented proteins were found in cellular (351 against 272 genes), developmental (102 against 53 genes), metabolic (135 against 115 genes), localization (82 against 68 genes), and homeostatic (16 against 10 genes) genes, as well as suppressed proteins in the immune system (seven against 27 genes). The functional implications of DEGs in corneal wound healing were analyzed (Fig. 5C). Genes upregulated in hCMFs were linked to molecular-function activity (55 against 39), structural activity (16 against 4), transporter activity (44 against 38), and cytoskeletal motor activity (five against three). The involvement of up- and downregulated genes in TGFβ-induced transdifferentiation of hCSFs to hCMFs was determined (Fig. 5D). The upregulated genes were connected to cellular anatomic entity (486 against 415 genes) and protein-containing complex (75 against 63 genes).

The tree plot from the Reactome database exhibited hierarchical organization and connections between enriched pathways (Fig. 6^7^). We observed changes in the interacting insulin-like growth factors, collagen assembly biosynthesis chain, activation of DNA ATR complex, cyclin A/B1/B2–associated events, and amplification from kinetochores signal genes (Fig. 6A). The dot plot revealed changes in pathways linked to the ECM, cell-cycle checkpoints, degradation of the ECM, and collagen formation (Fig. 6B). The gene clusters and combined network of co-modulated genes from the enriched pathways are shown in Figure 6C.

**Figure 6.**
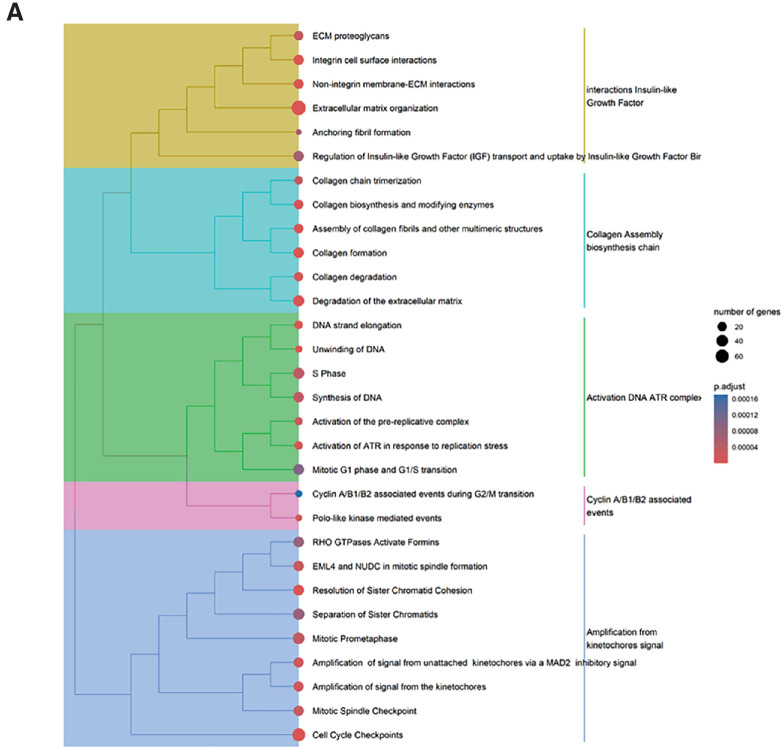
Pathway enrichment analysis using the Reactome database. (**A**) Tree plot of enriched pathways that illustrates the relationships between various biological pathways; the size of the nodes corresponds to the significance of the enrichment. Pathways are colored according to their enrichment scores, providing a visual representation of the key pathways involved in the DGE analysis. (**B**) In this dot plot, the *x*-axis (gene ratio) indicates the proportion of genes associated with each pathway relative to the total number of genes analyzed. The *y*-axis shows the names of the enriched pathways. The color gradient of the dots represents the adjusted adjusted *P* values, with *blue* indicating higher significance and *red* indicating lower significance. The size of the dots corresponds to the gene count involved in each pathway, with larger dots representing higher counts. (**C**) Network clusters showing genes involved in various ECM-related processes. The gene clusters show the genes and their interactions in five categories: ECM organization, degradation of the ECM, Assembly of collagen fibrils and other multimeric structures, non-integrin membrane–ECM interactions, and collagen biosynthesis and modifying enzymes. The integrated network combines these categories, with *node size* representing the number of connections (degree) and *node color* indicating fold change (*blue* for higher values, and *red* for lower values).

**Figure 6.**
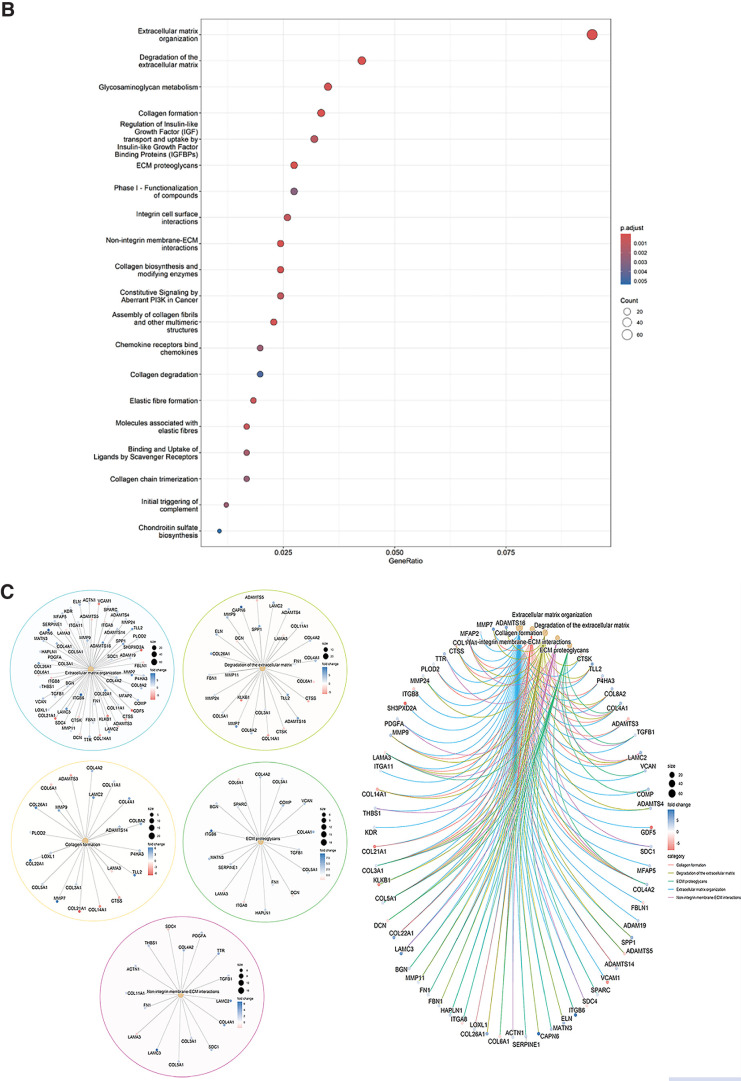
Continued.

**Figure 7.**
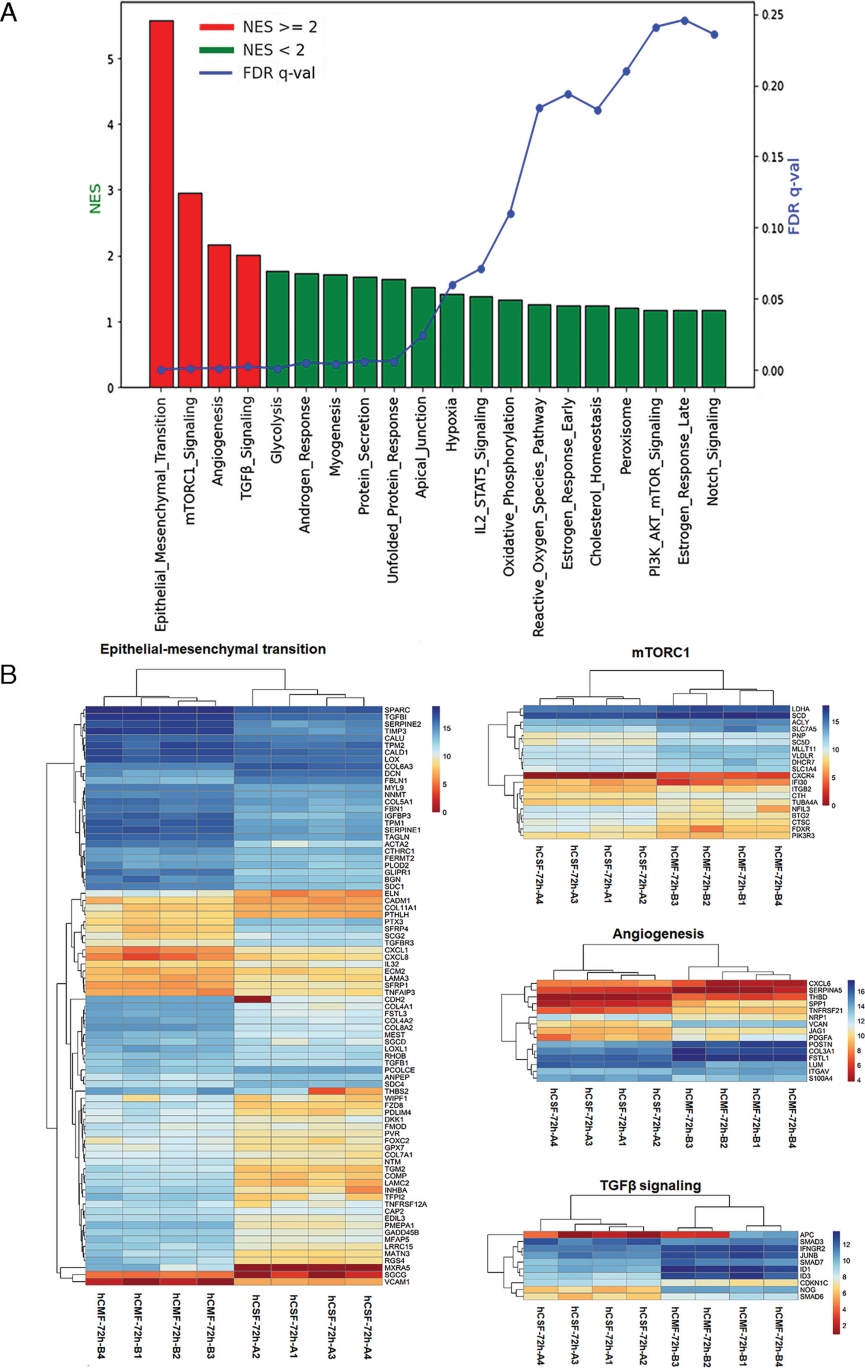
(**A**) Graphical illustration of gene set enhancement in various pathways. The most significant pathways with NES ≥ 2 and FDR *q* ≤ 0.05 are shown in *red*, and pathways with NES < 2 and/or FDR *q* > 0.05 are shown in *green*. The FDR *q* values of each pathway are shown in *blue*. (**B**) Heatmaps for the top four enriched pathways involving the epithelial–mesenchymal transition, mTORC1, angiogenesis, and TGFβ signaling across different samples are shown. The color gradient from *blue* to *red* indicates increasing gene expression levels.

The GSEA identified significant enrichment of several pathways (Fig. 7). The four most significantly enriched pathways (red) were epithelial–mesenchymal transition (EMT), with normalized enrichment score (NES) = 5.569 and false discovery rate (FDR) *q* = 0.000; mechanistic target of rapamycin complex 1 (mTORC1), with NES = 2.949 and FDR *q* = 0.000; angiogenesis, with NES = 2.176 and FDR *q* = 0.002); and TGFβ signaling, with NES = 2.008 and FDR *q* = 0.000. Pathways with NES < 2.0 and FDR *q* < 0.25 (green) were linked to glycolysis, androgen response, myogenesis, protein secretion, unfolded protein response, apical junction, hypoxia, IL-2/STAT signaling, oxidative phosphorylation, estrogen response early score, cholesterol homeostasis, peroxisome, PI3k/AKT signaling pathway, late estrogen response, and Notch signaling (Fig. 7A). The heatmaps for the top four enriched pathways across the hCSFs and hCMFs are presented in Figure 7B.

**Figure 8.**
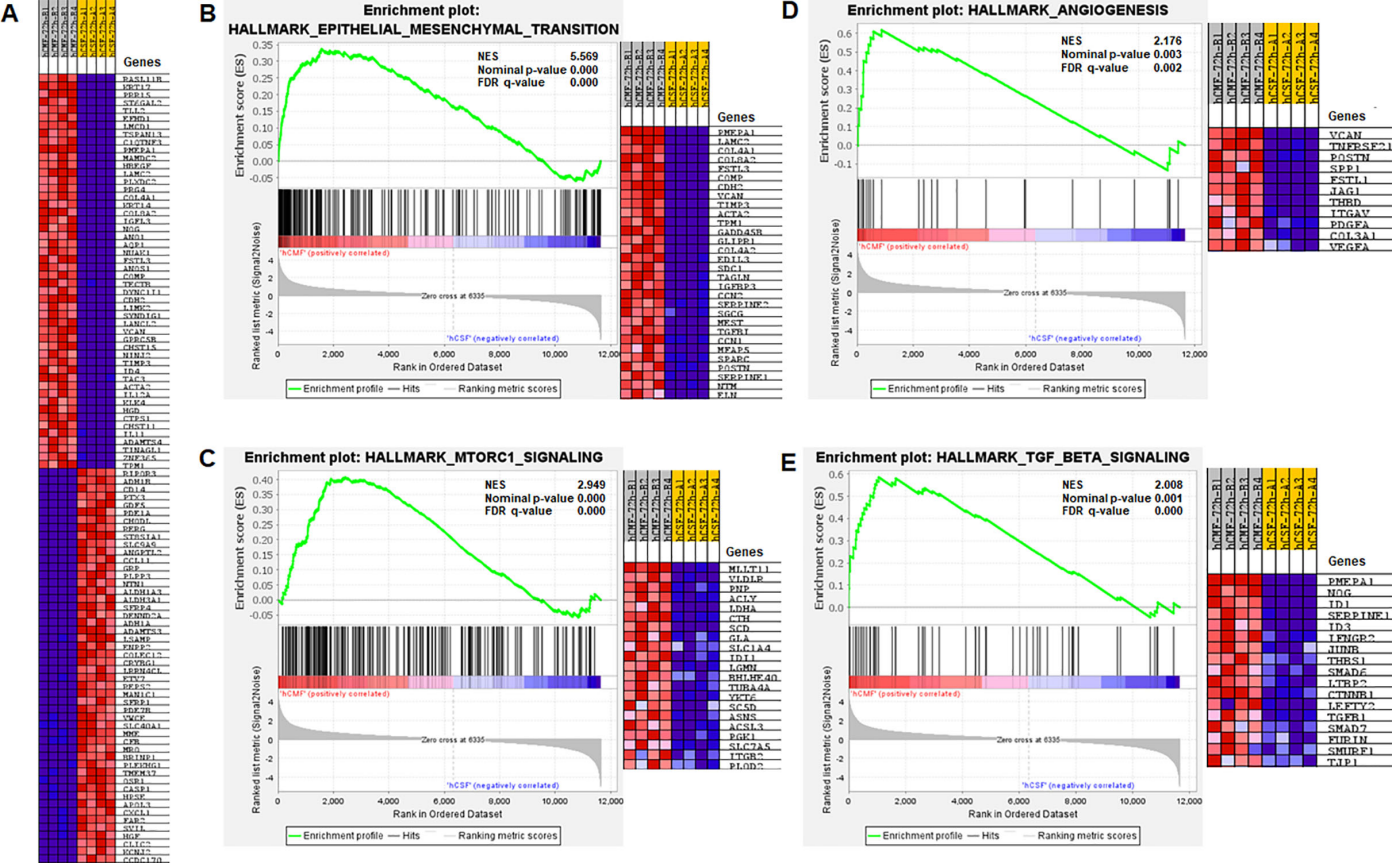
Pathway enrichment analysis of hCMFs versus hCSFs. (**A**) Heatmap of the top 50 genes in each cell line, with the red-to-blue color scale indicating higher to lower gene expression. (**B**–**E**) Significantly enriched pathways: EMT pathway (NES = 5.569), mTORC1 signaling pathway (NES = 2.949), angiogenesis (NES = 2.176), and TGFβ signaling (NES = 2.008). These pathways highlight a shift toward increased cellular differentiation and fibrotic responses in hCMFs, suggesting potential impacts on corneal fibrosis and transparency.

The GSEA heatmap of the top 50 genes in hCSFs and hCMFs is presented in Figure 8A, with a red and blue color gradient (where red indicates higher and blue indicates lower expression). The most remarkably affected genes during corneal wound healing belonged to the EMT (Fig. 8B), mTORC1 signaling (Fig. 8C), angiogenesis (Fig. 8D), and TGFβ signaling pathways (Fig. 8E).

### PPI Network Analysis

**Figure 9.**
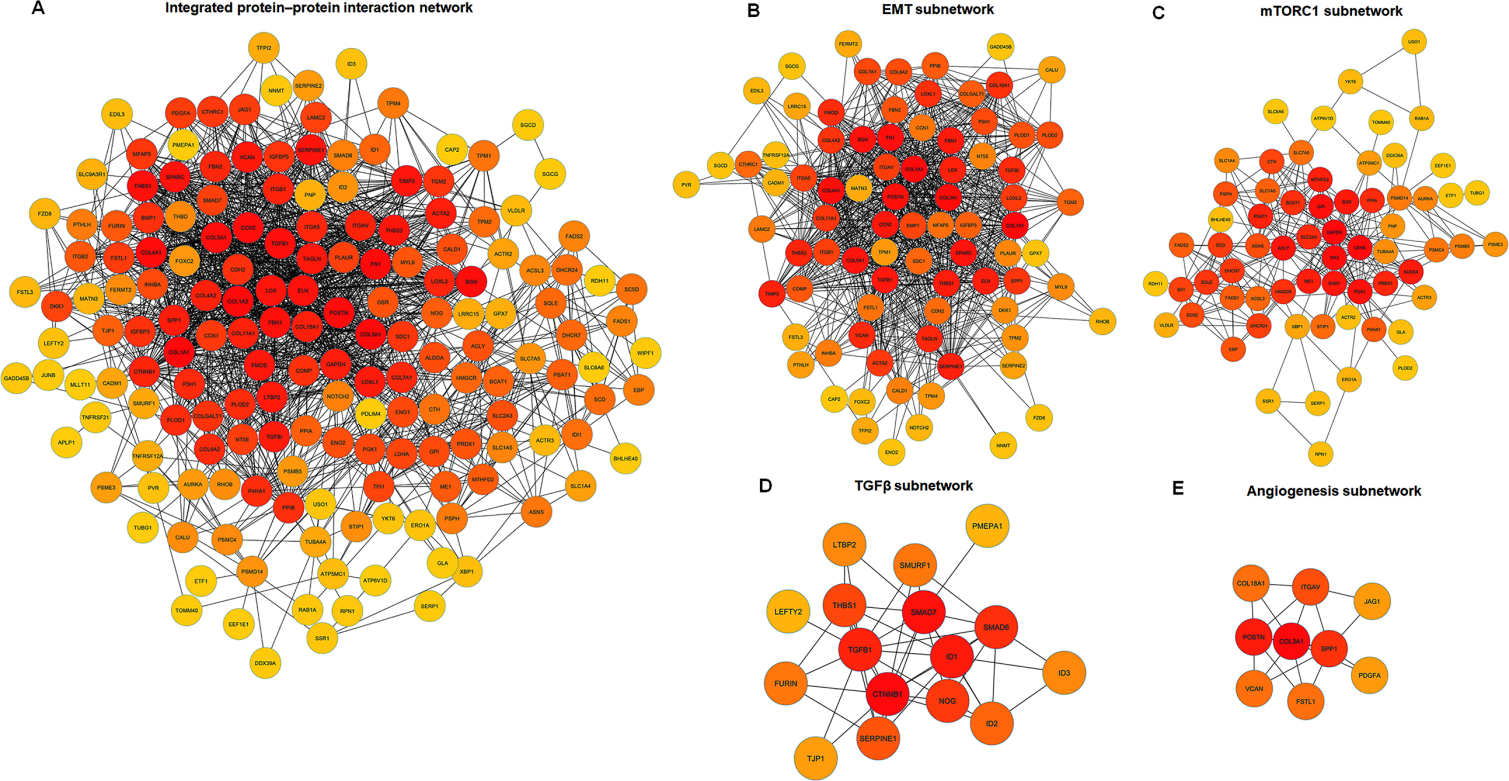
PPI networks. (**A**) Integrated protein‒protein interaction network of significantly enriched pathways in hCMFs. (**B**–**E**) Subnetworks of the four pathways related to the genes exhibiting the most significant enrichment: EMT (**B**), mTORC1 signaling (**C**), TGFβ signaling (**D**), and angiogenesis (**E**), derived from GSEA using gene counts. Influential nodes, as determined by the MCC algorithm using CytoHubba in Cytoscape, are emphasized to indicate their central role in the network (*red* to *yellow* color scale, with *red* indicating the most influential nodes).

The PPI networks demonstrating intricate interactions and functional convergence of signaling pathways in hCSFs and hCMFs were created using Cytoscape (Supplementary Fig. S3). The maximal clique centrality (MCC) algorithm within cytoHubba, a Cytoscape plugin, examined complexity and identified the most influential nodes in PPIs. The MCC algorithm pinpointed crucial nodes within a network based on their connectivity and position within maximal cliques and revealed the nodes within the network that are important in corneal wound healing (Fig. 9A). The EMT subnetwork showed proteins such as CCN2, ACTA2, COL4A1, and TIMP3 (Fig. 9B), the mTORC1 subnetwork showed MTOR, THBS1, and SERPINE1 proteins (Fig. 9C), and the TGFβ subnetwork showed signaling proteins including TGFB1, SMAD3, SMAD7, and CTNNB1(Fig. 9D). Also, the angiogenesis subnetwork exposed proteins such as PDGFA, VCAN, and SPP1, which are essential for new blood vessel formation (Fig. 9E). The integrated data from the significantly enriched pathways (EMT, mTORC1, TGFβ, and angiogenesis) are exhibited in Supplementary Table S2. Details regarding the nodes and edges of the integrated PPI network are provided in Supplementary Table S3. The top 20 influential nodes revealed by the network analysis are shown in Table 4.

**Table 4.** Influential Nodes With a Possible Role in Corneal Wounds and Their Ranking in the Network

Protein Name	Node Rank	Gene Fold Change	Role in Cornea
Type I collagen alpha-1 (COL1A1)	1	2.27	COL1A1 is a member of group I of the collagen family, noted for its fibril-forming capability, essential for maintaining the structural integrity and transparency of the corneal stroma.^30^
Fibronectin (FN1)	2	2.93	FN1 facilitates adherence to cell surfaces and interaction with key components such as collagen, fibrin, heparin, DNA, and actin. It supports cell adhesion, movement, opsonization, and wound healing and maintains cellular integrity, which is crucial for the health and functionality of the cornea.^31^
Collagen type I alpha 2 (COL1A2)	3	2.26	COL1A2 is recognized for its ability to form fibrils and is categorized within the broader group of fibrillar collagens essential for the structural framework and transparency of the corneal tissue.^32^
Collagen type III alpha 2 (COL3A1)	4	3.29	COL3A1 is typically present in soft connective tissues alongside type I collagen and is crucial for the corneal structure, contributing to its integrity and function.^33^
Periostin (POSTN)	5	5.22	POSTN plays an important role in enhancing cell attachment and dispersion. It aids in incorporating BMP1 into the fibronectin matrix, a vital step for activating lysyl oxidase LOX through proteolysis.^34^ It has unique expression patterns and functional roles in maintaining the properties of human limbal stem cells.^35^ This action may play an essential role in improving the corneal structure and its overall function.
Actin alpha-2, α-smooth muscle actin 2 (ACTA2)	6	16.63	αSMA is a hallmark of corneal wound healing and fibrosis and signifies the transdifferentiation of fibroblasts into myofibroblasts in response to TGFβ1 injury, treatment, and surgical intervention.^36^
Biglycan (BGN)	7	5.91	BGN is a small leucine-rich proteoglycan known to play a significant role in the structure and function of the ECM in various tissues, including the cornea. In the context of the cornea, BGN may be involved in collagen fiber assembly, contributing to the regulation of corneal transparency and biomechanical properties.^37^
Cellular communication network factor 2 (CCN2)	8	4.11	CCN2, or connective tissue growth factor (CTGF), promotes the proliferation and differentiation of keratocytes, the principal cells of the corneal stroma. By mediating cell adhesion, CCN2 facilitates the interaction between corneal cells and the ECM, crucial for wound healing. Additionally, its ability to enhance fibroblast growth factor–induced DNA synthesis suggests a role in corneal cell regeneration and repair following injury.^38^
Transforming growth factor β1 (TGFβ1)	9	4.20	TGFβ1 binds to its receptors (TGFBR1 and TGFBR2), initiating cellular signaling pathways that regulate various cell functions. TGFβ1 promotes keratocyte proliferation and differentiation, collagen production, and EMT processes.^39^
Collagen type V alpha 1 (COL5A1)	10	3.59	COL5A1 is a fibrillar-forming collagen. The interactions of type V collagen with molecules such as DNA, heparan sulfate, thrombospondin, heparin, and insulin suggest a multifaceted role in cellular functions, including cell adhesion, migration, and a possibly influence on corneal healing processes.^40^
Thrombospondin 1 (THBS1)	11	4.93	THBS1 is an adhesive glycoprotein crucial for mediating interactions both between cells and between cells and the ECM. The role of THBS as a ligand for CD36 is particularly significant in the cornea for its antiangiogenic properties, helping maintain the avascular nature of the cornea essential for transparency and vision.^41^
Fibrillin 1 (FBN1)	12	3.76	FBN1 is a structural component of the ECM and forms part of the microfibrils that not only provide structural support but also play a regulatory role in the corneal tissue. These microfibrils, independent of elastin, contribute significantly to the tensile strength of the cornea and provide anchoring functions. FBN1 interacts with various growth factors, including bone morphogenetic proteins (BMPs) and latent transforming growth factor-beta-binding proteins (LTBPs), alongside cell-surface integrins and other ECM components.^42^
Elastin (ELN)	13	17.60	ELN is less prominent but plays a crucial role due to the unique requirements of the cornea for transparency and mechanical stability. Although elastin is a major structural protein in tissues that undergo significant expansion and recovery, its presence in the cornea is relatively minimal. However, the elastin that does exist in the cornea contributes to its ability to withstand mechanical stress and maintain its shape and structure. This is particularly important in the peripheral corneal regions and the limbus, where slight elasticity might help in accommodating intraocular pressure changes and ensuring the integrity of the ocular surface.^43^
Secreted protein acidic and rich in cysteine (SPARC)	14	3.28	SPARC regulates cellular behavior and maintains ECM composition through its interactions with cytokines and various components of the matrix. This multifunctional glycoprotein binds to essential molecules such as calcium, copper, diverse collagen types that are critical for corneal structure, albumin, thrombospondin (which supports cell–matrix interactions), platelet-derived growth factor (PDGF), and cell membranes.^44^
Protein-lysine 6-oxidase (LOX)	15	2.20	LOX plays a crucial role in the post-translational modification of collagen and elastin precursors, which are essential components of the corneal stroma. This enzyme catalyzes the oxidative deamination of peptidyl lysine residues, a critical step in the cross-linking process that strengthens collagen and elastin fibers.^45^
Serine protease inhibitor E1 (SERPINE1)	16	5.91	SERPINE1 acts as a serine protease inhibitor with a critical role in modulating fibrinolysis and cell migration. It primarily inhibits tissue-type (PLAT) and urokinase-type (PLAU) plasminogen activators, thereby controlling the degradation of fibrin clots and regulating cell adhesion and spreading.^46^^,^^47^
Tissue inhibitor of metalloproteinase 3 (TIMP3)	17	8.05	TIMP3 irreversibly inactivates metalloproteinases, including collagenases, through binding to their catalytic zinc cofactor. This action helps regulate corneal remodeling and healing processes by inhibiting enzymes such as MMP-1, MMP-2, MMP-3, MMP-7, MMP-9, MMP-13, MMP-14, and MMP-15, which are involved in the breakdown of the ECM.^48^
Thrombospondin 2 (THBS2)	18	Not significant	THBS2 is an adhesive glycoprotein essential for facilitating interactions both between cells and between cells and the ECM. It serves as a ligand for CD36, playing a significant role in mediating antiangiogenic effects.^49^
Transgelin (TAGLN)	19	4.29	TAGLN acts as an actin cross-linking/gelling protein contributing to the structural organization and contractile properties in non-ocular tissues.^50^ Its role in corneal wound healing is not known.
Fibromodulin (FMOD)	20	2.32	FMOD plays a key role in regulating the formation of collagen fibrils. As a member of the small leucine-rich proteoglycan (SLRP) family, specifically within the SLRP class II subfamily, it significantly contributes to collagen fibrillogenesis, impacting the mechanical properties and overall health of the cornea.^51^

### qRT-PCR for Selected Genes

**Figure 10.**
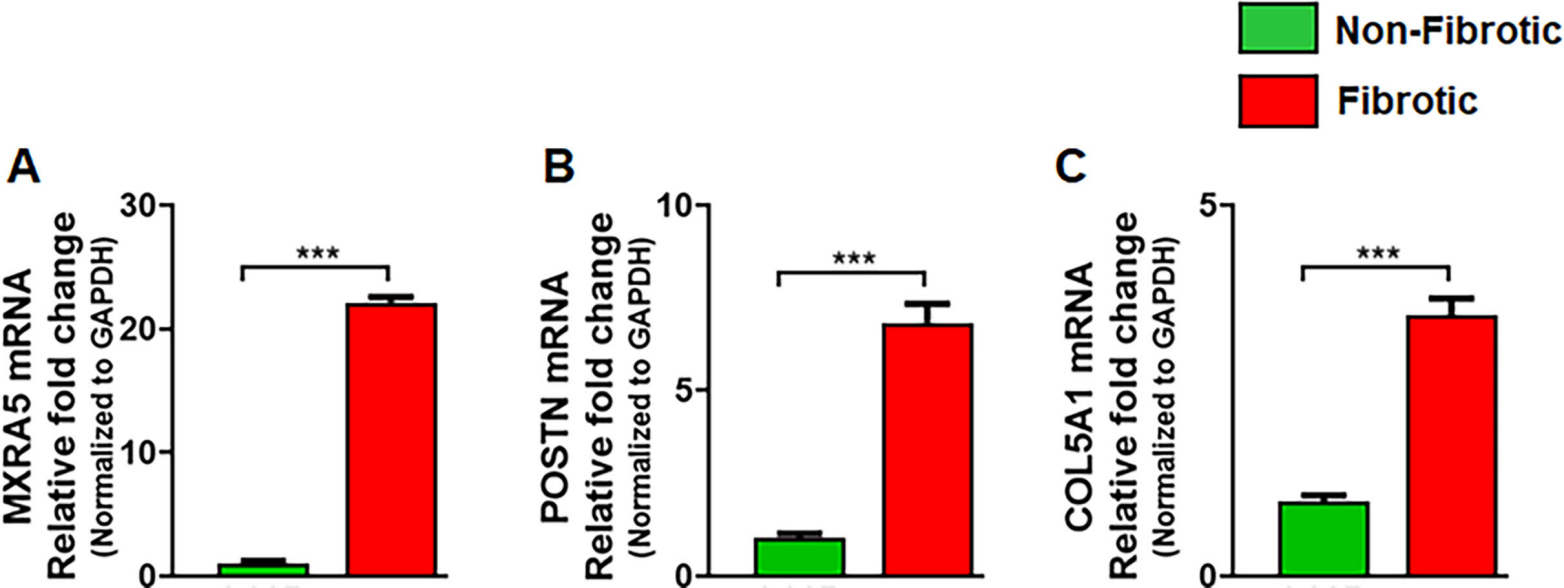
(**A**–**C**) Real-time PCR quantification detected significant (*P* < 0.001) increases in the gene expressions of MXRA5 (**A**), POSTN (**B**), and COL5A1 (**C**) in fibrotic (hCMF) cells compared to non-fibrotic (hCSF) cells as found in RNA-seq data analysis.

The DGE analysis identified expression of the *MXRA5* gene in corneal stromal cells, hCSFs and hCMFs. Levels of the *MXRA5* gene were significantly upregulated in hCMFs compared to hCSFs (*P* < 0.0001) (Table 5). The elevated *MXRA5* expression was further confirmed with qRT-PCR (22.11 ± 0.45-fold; *P* < 0.0001) (Fig. 10A). Likewise, the expression of *POSTN* and *COL5A1* genes, whose functional role has been poorly studied in the human cornea, was also identified. The qRT-PCR confirmed significant upregulation of *POSTN* (6.83 ± 0.51-fold; *P* = 0.0001) (Fig. 10B) and *COL5A1* (3.52 ± 0.22-fold; *P* < 0.0001) (Fig. 10C) in hCMFs compared to hCSFs.

**Table 5.** Tope Ten Upregulated and Downregulated Differentially Expressed Genes

DGE	Gene ID	Name	log_2_(FC)	*P*, Adjusted
Upregulated	*MXRA5*	Matrix-remodeling associated 5	28.088	6.46E-15
	*TSPAN2*	Tetraspanin 2	12.848	3.64E-15
	*KIAA1644/ZNF638*	Zinc finger protein 638	11.165	1.24E-04
	*C4orf26*	Chromosome 4 open reading frame 26	10.905	4.28E-09
	*WNT4*	Wnt family member 4	10.686	1.37E-18
	*DNM3OS*	DNM3 opposite strand	10.193	5.16E-12
	*LINC01929*	Long intergenic non-protein coding RNA 1929	9.9123	1.68E-15
	*KLK4*	Kallikrein related peptidase 4	9.763	1.20E-17
	*LPAR5*	Lysophosphatidic acid receptor 5	9.700	3.55E-16
	*CAPN6*	Calpain 6	9.634	4.88E-15
Downregulated	*C10orf105*/*DMXL2*	Dmx like 2	−10.147	3.55E-18
	*RIPOR3*	Rho family interacting cell polarization regulator 3	−9.902	1.41E-19
	*KIT*	KIT proto-oncogene, receptor tyrosine kinase	−9.330	1.61E-13
	*ABCA9*	ATP binding cassette subfamily A member 9	−9.040	3.29E-13
	*TXLNB*	Taxilin β	−9.003	8.44E-13
	*ADH1B*	Alcohol dehydrogenase 1B (class I)	−8.335	5.76E-30
	*ZBTB16*	Zinc finger and BTB domain containing 16	−8.289	1.86E-11
	*SCN4B*	Sodium voltage-gated channel beta subunit 4	−8.094	1.20E-11
	*GDF5*	Growth differentiation factor 5	−8.004	4.79E-30
	*SH3PXD2A*	SH3 and PX domains 2A	−7.881	1.16E-03

### MXRA Protein Expression in Human Cornea

**Figure 11.**
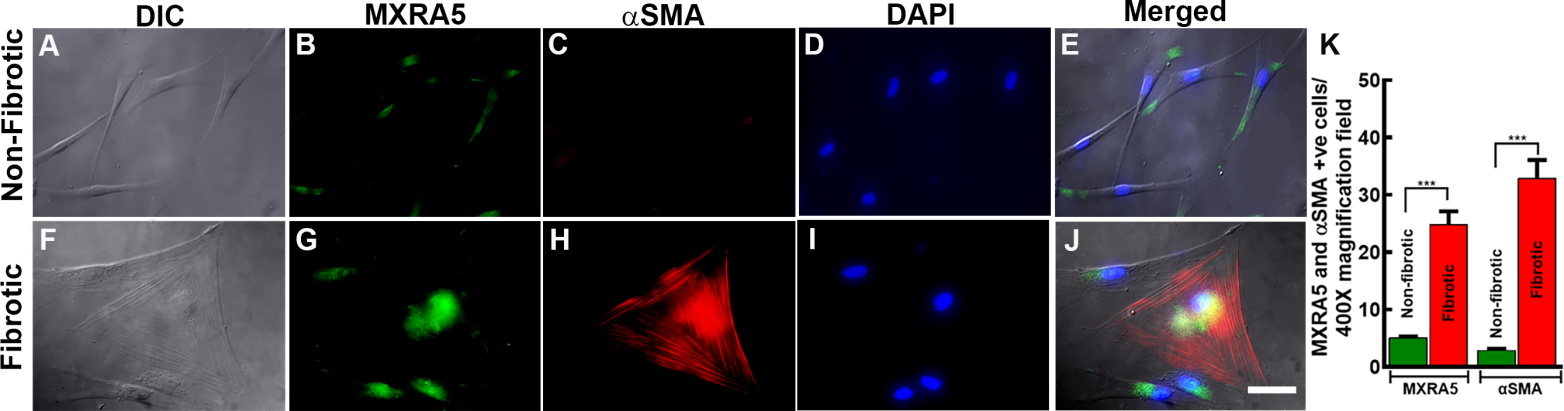
Double immunofluorescence staining results for αSMA and MXRA5 in non-fibrotic and fibrotic corneal stromal cells. (**A**–**E**) Non-fibrotic cells. (**F**–**J**) Fibrotic cells. Panel **A** shows a differential interference contrast (DIC) image depicting the general morphology of non-fibrotic cells, and panel **F** shows fibrotic cells. Panels **B** and **G** display the immunofluorescence images for αSMA (*red*), which is absent in non-fibrotic cells but clearly expressed in fibrotic cells. Panels **C** and **H** show the immunofluorescence images for MXRA5 (*green*), indicating its expression in both cell types but with higher expression in fibrotic cells. Panels **D** and **I** display DAPI staining (*blue*) marking the nuclei of the cells. Panels **E** and **J** are merged images combining αSMA, MXRA5, and DAPI. The comparison highlights the elevated expression of MXRA5 in fibrotic cells, as indicated by the more intense *green fluorescence* in panels **H** and **J**. (**K**) The quantification revealed significant (*P* < 0.001) increase in expression of MXRA5 and αSMA in corneal stromal fibrotic cells compared to non-fibrotic cells. *Scale bar*: 40 µm.

**Figure 12.**
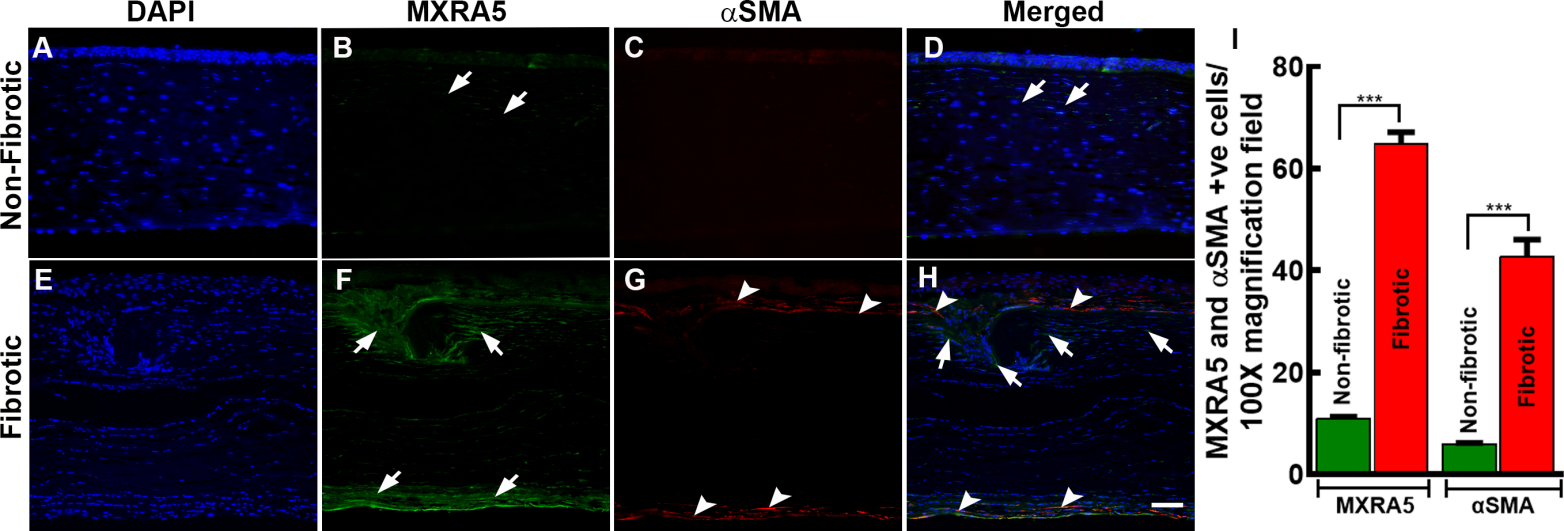
Immunofluorescence staining of non-fibrotic and fibrotic corneal tissues from human subjects. (**A**–**D**) Non-fibrotic tissue with DAPI staining for nuclei (*blue*, **A**), MXRA5 staining (*green*, **B**, *arrows*), minimal αSMA staining (*red*, **C**), and a merged image (**D**) highlighting colocalization of MXRA5 with nuclei (*arrows*). (**E**–**H**) Fibrotic tissue with DAPI staining (*blue*, **E**), increased MXRA5 expression (*green*, **F**, *arrows*), enhanced αSMA expression (*red*, **G**, *arrowheads*), and a merged image (**H**) showing colocalization and increased expression of MXRA5 (*arrows*) and αSMA (*arrowheads*) in fibrotic regions. (**I**) The quantification revealed a significant (*P* < 0.001) increase in MXRA5 and αSMA positive cells in human corneal fibrotic tissue compared to non-fibrotic tissue. *Scale bar*: 100 µm.

Double immunofluorescence displaying co-localization of αSMA (red) and MXRA5 (green) in the cytoplasm of hCMFs (Figs. 11F–J) but not in hCSFs (Figs. 11A–E) revealed involvement of MXRA5 protein in corneal fibrosis, as well as co-localization of αSMA (red) and MXRA5 (green) protein expression in donor fibrotic human cornea (Figs. 12E–H) and its absence in normal (non-fibrotic) human cornea (Figs. 12A–D). Furthermore, these data revealed involvement of MXRA5 in corneal fibrosis.

## Discussion

This RNA-seq study identified 20,269 gene annotations and significantly modulated 3843 DGEs, 816 upregulated genes, 739 downregulated genes, four critically enriched pathways, and PPI networks in hCSFs and hCMFs, thus providing valuable new insights into molecular events during corneal wound healing. Additionally, this study, for the first time to the best of our knowledge, to the best of our knowledge, identified expression of the *MXRA5* gene in human corneal stroma and its role in corneal fibrosis and the involvement of *POSTN* and *COL5A1* genes in human corneal stromal cell modulation.

The non-ocular literature reveals the expression and functional role of *MXRA5* in TGFβ1-driven fibrotic processes and ECM modulation.^52^^–^^54^ This study detected low *MXRA5* mRNA and protein expression in hCSFs and normal human cornea but significantly increased levels in hCMF and fibrotic cornea (Figs. 11, 12), thus revealing its role in corneal fibrosis development. Furthermore, this novel observation led to the suggestion that *MXRA5* can potentially serve as an attractive target to regulate TGFβ1-induced pathological events in the cornea. The specific function of *MXRA5* in corneal wound healing is still unknown. Apart from the *MXRA5*, increased expression of *POSTN* (rank 5) and *COL5A1* (rank 10) genes was observed in hCMFs during RNA-seq analysis and qRT-PCR (Table 4, Fig. 10). *POSTN* regulates cell attachment/dispersion, fibronectin matrix, lysyl oxidase, and limbal stem cells.^34^^,^^35^ Such reports suggest that POSTN may play a role in maintaining corneal integrity; however, its functional role in corneal healing is still poorly known. Likewise, the functional role of COL5A1, a fibrillar-forming collagen, in corneal stroma remains elusive, despite the fact that it can interact with heparan sulfate, thrombospondin, and heparin and influence cell adhesion and migration.^40^ Furthermore, the current study found significant changes in *WNT4*, *αSMA*/*ACTA2*, *AQP1*, *NOX4*, *TLL2*, *LMCD1*, *PMEPA1*, *LANCL2*, *PTX3*, and many other genes (Fig. 8, Table 4) that are shown to affect TGFβ1 signaling, myofibroblast formation, reactive oxygen species production, inflammation, or ECM formation/remodeling in several non-ocular tissues^55^^–^^60^; however, their precise role in corneal stromal healing has not yet been fully explored.

Another notable finding of this RNA-seq study is exposing a dominant role of genes associated with EMT, mTORC1 signaling, TGFβ signaling, and angiogenesis in corneal stromal fibroblast differentiation to myofibroblast (Figs. 5–8). These data highlight a distinct shift in cellular mechanisms and signaling pathways during myofibroblast formation, consistent with earlier reports. Also, the PPI networks constructed from these enriched pathways show interactions and functional convergence of the pathways, serving as a testament to the complexity of corneal wound healing and highlighting the multifaceted roles of various signaling pathways in this process.

This study provides valuable new information regarding genes linked to corneal fibroblast differentiation, but it has some limitations. These include not fully addressing the impact of biological variables such as sex, age, and race; usage of human corneas from donors between 46 and 76 years old; a single dose of TGFβ1; primary cultures generated from multiple donors; and the use of 10% fetal bovine serum to obtain primary hCSF cultures. Our future studies will address these limitations by employing a larger and more diverse cohort of donors from different demographics. Also, additional research employing a broader range of TGFβ1 concentrations and serum conditions will help to better understand the spectrum of corneal stromal cellular responses in corneal wound healing and fibrosis.

In summary, this study provides a comprehensive analysis of gene count, DGE, pathways, PPI networks, and the top genes influencing corneal fibroblast differentiation to myofibroblasts. The genetic landscape governing the differences between corneal fibroblasts and myofibroblasts offers a deeper understanding of molecular dynamics of corneal wound healing and suggests potential targets for developing strategies to treat corneal fibrosis and restore vision.

## Supplementary Material

Supplement 1

Supplement 2

Supplement 3

Supplement 4
