# Supplementary material for: RNA-Seq Analysis Unraveling Novel Genes and Pathways Influencing Corneal Wound Healing
Source: Invest Ophthalmol Vis Sci. 2024 Sep 6;65(11):13. doi: 10.1167/iovs.65.11.13 (PMC11383191; doi:10.1167/iovs.65.11.13)
Supplement: Supplement 1 [file iovs-65-11-13_s001.pdf]

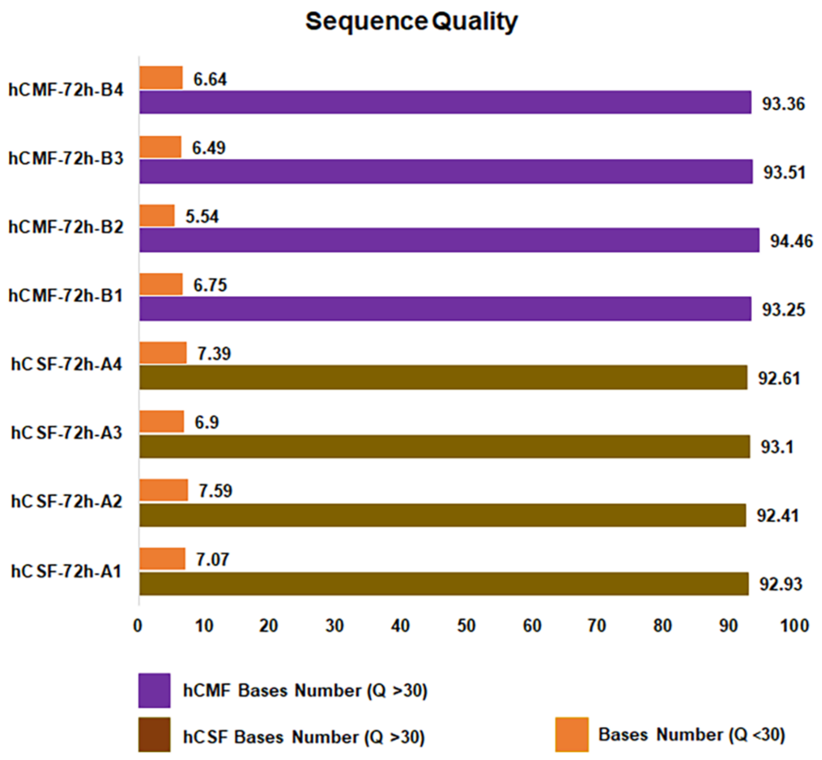

FIGURE S1. Graphical representation of the quality scores of the hCMF and hCSF sequences. The percentage of the number of bases with Q>30, showing good quality across the sequences.

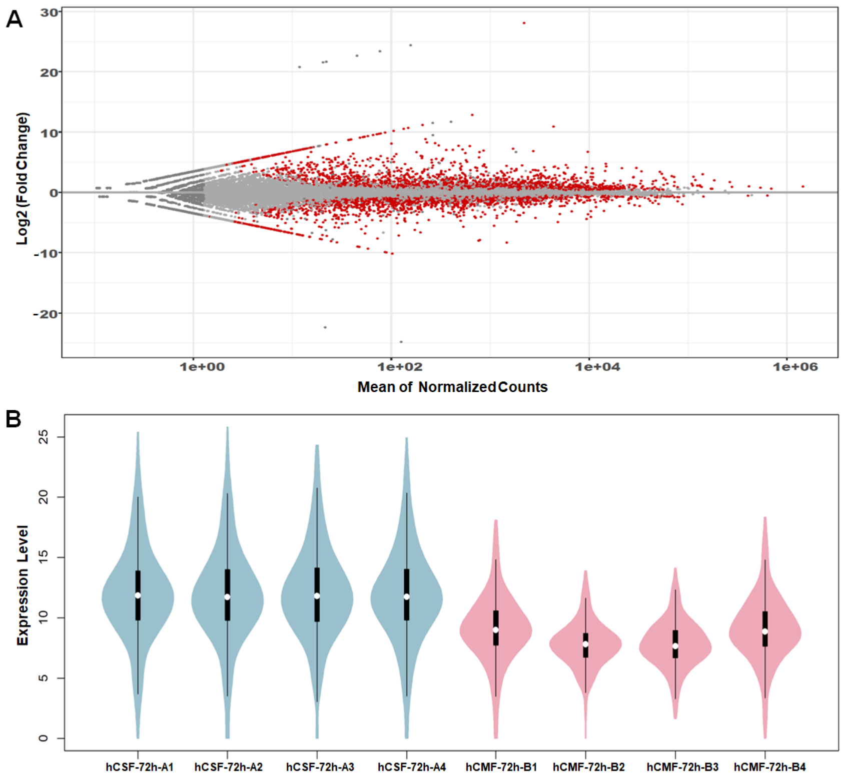

FIGURE S2. Comparative analysis of gene expression in the hCMF and hCSF types. (A) MA plot of the log2(FC) (M-value) and average expression (A-value) showing changes in gene expression in hCMF compared to hCSF. (B) Violin plot showing difference in gene expression level between the hCMF and hCSF cell types.

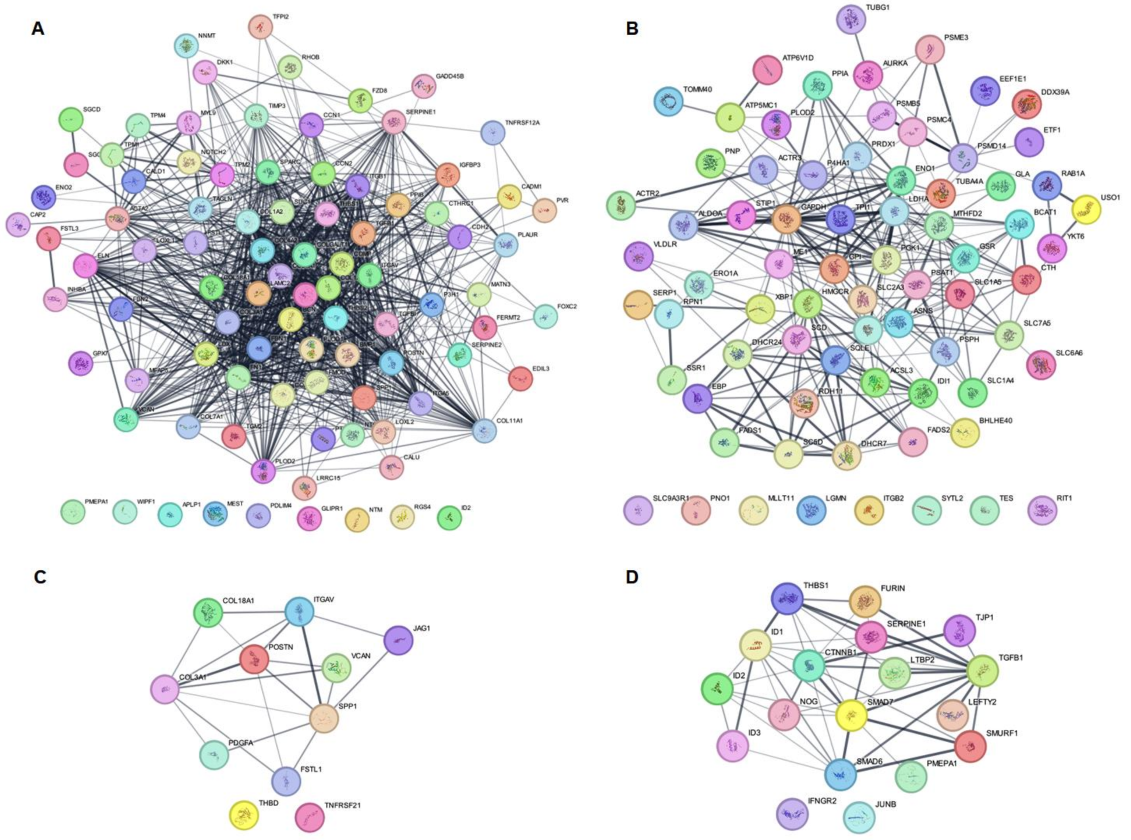

FIGURE S3. The protein networks for four critical signaling pathways based on core enrichment data from gene set enrichment analysis. (A) EMT network depicting key proteins and interactions involved in epithelial-mesenchymal transition. (B) mTORC1 signaling network, showing protein that may be involved in the regulation of cellular growth and metabolism. (C) Angiogenesis network, emphasizing proteins that mediate new blood vessel formation, crucial for tissue growth and repair. (D) Showing the TGFβ signaling protein network.
